# Supplementary material for: Global gridded population datasets systematically underrepresent rural population
Source: Nat Commun. 2025 Mar 18;16:2170. doi: 10.1038/s41467-025-56906-7 (PMC11920052; doi:10.1038/s41467-025-56906-7)
Supplement: Supplementary file 1 — Supplementary Information [file 41467_2025_56906_MOESM1_ESM.pdf]

Supplementary information for:

“Global gridded population datasets systematically underrepresent rural population”

Josias Láng-Ritter[1,2,*], Marko Keskinen[1], Henrikki Tenkanen[2]

[1] Water and Development Research Group, Department of Built Environment, Aalto University, Tietotie 1E, 02150 Espoo, Finland

[2] GIScience for Sustainability Transitions Lab, Department of Built Environment, Aalto University, Otakaari 4, 00076, Espoo, Finland

*Corresponding author; josias.lang-ritter@aalto.fi

Supplementary items

- **Supplementary Table 1.** Bias percentages by country.
- **Supplementary Table 2.** Statistical properties of the 307 investigated rural areas.
- **Supplementary Figure 1.** LandScan data for reference year 2000 in Tuyên Quang Province, Northern Vietnam.
- **Supplementary Figure 2.** Correlation analysis between country electrification and population estimation bias.
- **Supplementary Figure 3.** Sensitivity analysis of population dataset resolution.
- **Supplementary Figure 4.** Effect of UN-adjustment on population grids.
- **Supplementary Figure 5.** Results with omission of Chinese reservoirs.
- **Supplementary Figure 6.** Size distribution of the 307 investigated rural areas.
- **Supplementary Figure 7.** Results assuming 10–14 years of temporal offset.

Supplementary Table 1. Numeric values of bias percentages of the five population datasets by country, as illustrated in Figures 7 and 8 in the manuscript.

ISO3	Bias percentage						Reference years (number of areas evaluated)
	GWP	GRUMP	GHS-POP	LandScan	WorldPop	Mean	
ALB	-25.8		-96.4	22.9	-14.7	-28.5	2010 (1)
AUS		-98	-96.5			-97.2	1995 (1), 1985 (3), 1980 (4), 1975 (2)
AUT			-0.6			-0.6	1975 (1)
BFA	-76.1		-94.2	-79.7	-74.2	-81	2010 (1), 1985 (1)
BOL	-11.5		-99.6	-86	-73.4	-67.6	2010 (1)
BRA	-11.5	-24.2	-99.1	-53.3	-28.2	-43.3	2005 (5), 2000 (4), 1995 (8), 1990 (4), 1985 (1), 1980 (5), 1975 (3)
CHN	-64.8	-71.2	-82.2	-68.5	-52.1	-67.8	2010 (1), 2005 (13), 2000 (25), 1995 (32), 1990 (24), 1985 (26), 1980 (35), 1975 (47)
COL		-90.5	-97			-93.8	1995 (1), 1980 (4), 1975 (1)
CYP			-99.6			-99.6	1980 (1)
CZE		-19.7	-96.7			-58.2	1990 (1), 1985 (1), 1980 (1)
DEU			-87.6			-87.6	1985 (2), 1980 (1), 1975 (1)
FIN			-93.8			-93.8	1985 (1), 1980 (1), 1975 (2)
GEO			-99.5			-99.5	1980 (1)
GHA	509.1		-99.7	582.2	282	318.4	2005 (1)
GIN		-39	-100			-69.5	1990 (1)
KHM	3.4		-18.9	46.4	51.2	20.5	2010 (1)
LAO	-77.2		-85	-66.3	-62.5	-72.8	2005 (2)
LKA			-99			-99	1985 (1)
LSO		130	-91			19.5	1995 (1), 1990 (1)
MKD		573.7	28.6			301.2	1995 (1), 1975 (1)
MMR	-81.3		-99.6	-70.5	-78.9	-82.6	2010 (1)
MYS	-93.7		-99.8	-67.2	-90.3	-87.8	2005 (1)
NZL			-94.8			-94.8	1980 (1)
PAK	-72.8	-66.1	-99.9	-80.7	-51.6	-74.2	2000 (2), 1995 (1)
PAN	-94.5		-98.2	-81.7	-88.2	-90.6	2005 (1)
POL	-32.9	-41.3	-96.9	-78.4	-12	-52.3	2000 (1), 1995 (1), 1990 (1), 1985 (1), 1980 (4), 1975 (1)
ROU			-97.9			-97.9	1985 (2), 1980 (1), 1975 (1)
RUS	-84.9		-99	-91	-96.3	-92.8	2005 (1), 1980 (1)
SWE			-90			-90	1980 (1)
TGO			-99.8			-99.8	1980 (1)
TJK			-99.9			-99.9	1975 (1)
TZA			-95.2			-95.2	1975 (1)
URY			-96.7			-96.7	1975 (1)
VEN		5039.2	-96.4			2471.4	1990 (1), 1980 (1)
VNM	-20.8	-18.2	-93.9	79.9	70.5	3.5	2000 (1)

Supplementary Table 2. Statistical properties of the sample of 307 rural areas. Altitude data used represents the altitude of the bottom of dam structure (metres above mean sea level), as reported by ICOLD.

Characteristic of study sample	Mean	Median	Std	Min	Max
Area [km^2]	73.3	9.0	278.3	1.0	3 645.6
Population [people]	11 818.2	1 864.0	56 362.7	0.0	900 000.0
Population density [people / km^2]	318.8	219.9	337.8	0.0	1 476.6
Altitude [mamsl]	481.3	270.0	609.2	7.0	4 250.0

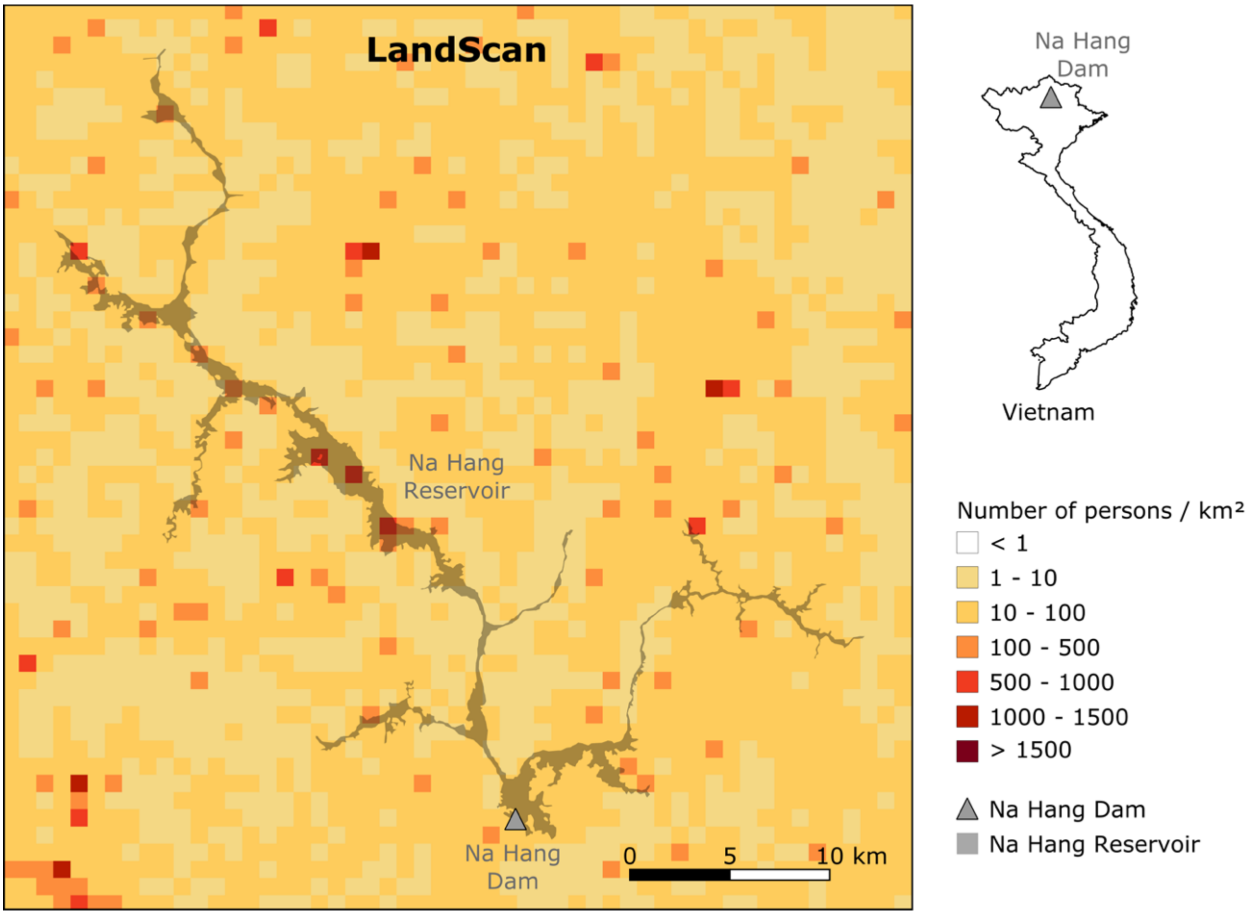

Supplementary Figure 1. Enlargement of parts of Figure 1d in the manuscript, showing LandScan population data of the reference year 2000 around Na Hang Dam and Reservoir in Northern Vietnam, completed in 2008 and displacing 4000 people. Note the data resolution of 1 km for this dataset, and that only the populations inside the portions of the 1-km-cells falling inside the grey polygon are taken into account in the analysis of this study (see Methods for details). Country boundary courtesy of ©EuroGeographics.

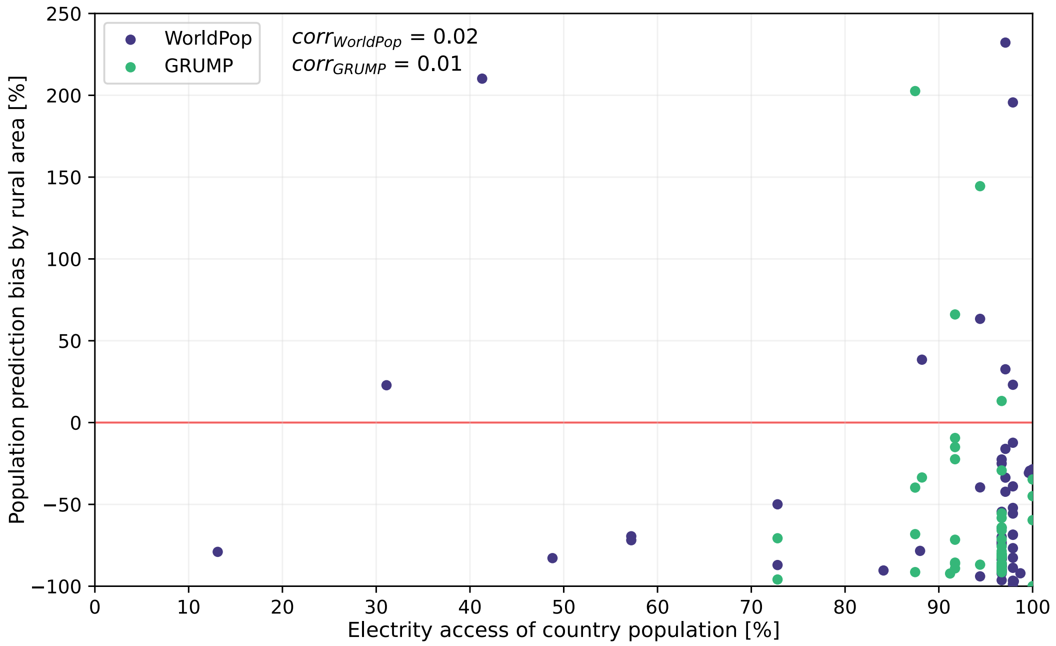

Supplementary Figure 2. Correlation analysis between country electrification and population estimation bias in rural areas, for WorldPop and GRUMP. Electrification data was unavailable for some countries and reference years, resulting in a reduced set of rural areas evaluated here ($N_{WorldPop} = 63$; $N_{GRUMP} = 48$). The figure shows that country electrification does not appear to have any significant influence on the accuracy of GRUMP and WorldPop, i.e. the two population datasets that use nighttime lights in their algorithms.

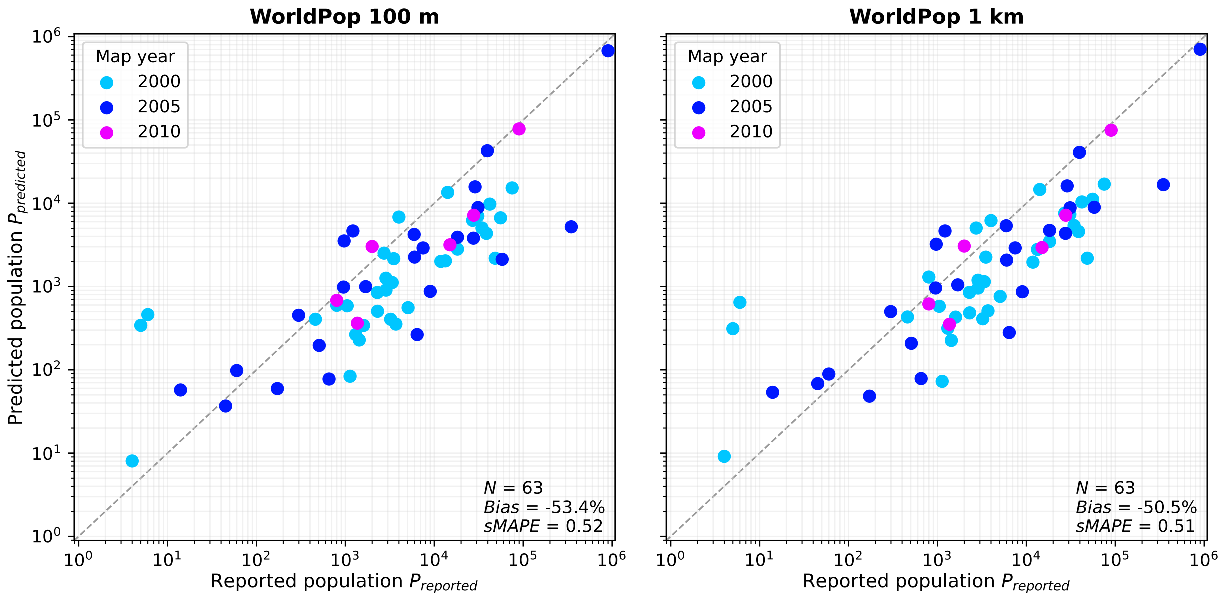

Supplementary Figure 3. Comparison of main results using WorldPop data in 100 m (left) and 1 km resolution (right). The Figure shows that using WorldPop in 1 km resolution leads to similar results as using the 100 m data (biases of –50.5% and –53.4%, respectively). Such marginal differences are common in studies combining gridded and vectorized data and often relate to the Modifiable Areal Unit Problem (MAUP). This similarity of the results using WorldPop in different resolutions indicates that the assumption of an even population distribution within the data cells does not significantly affect the biases we identified in this study.

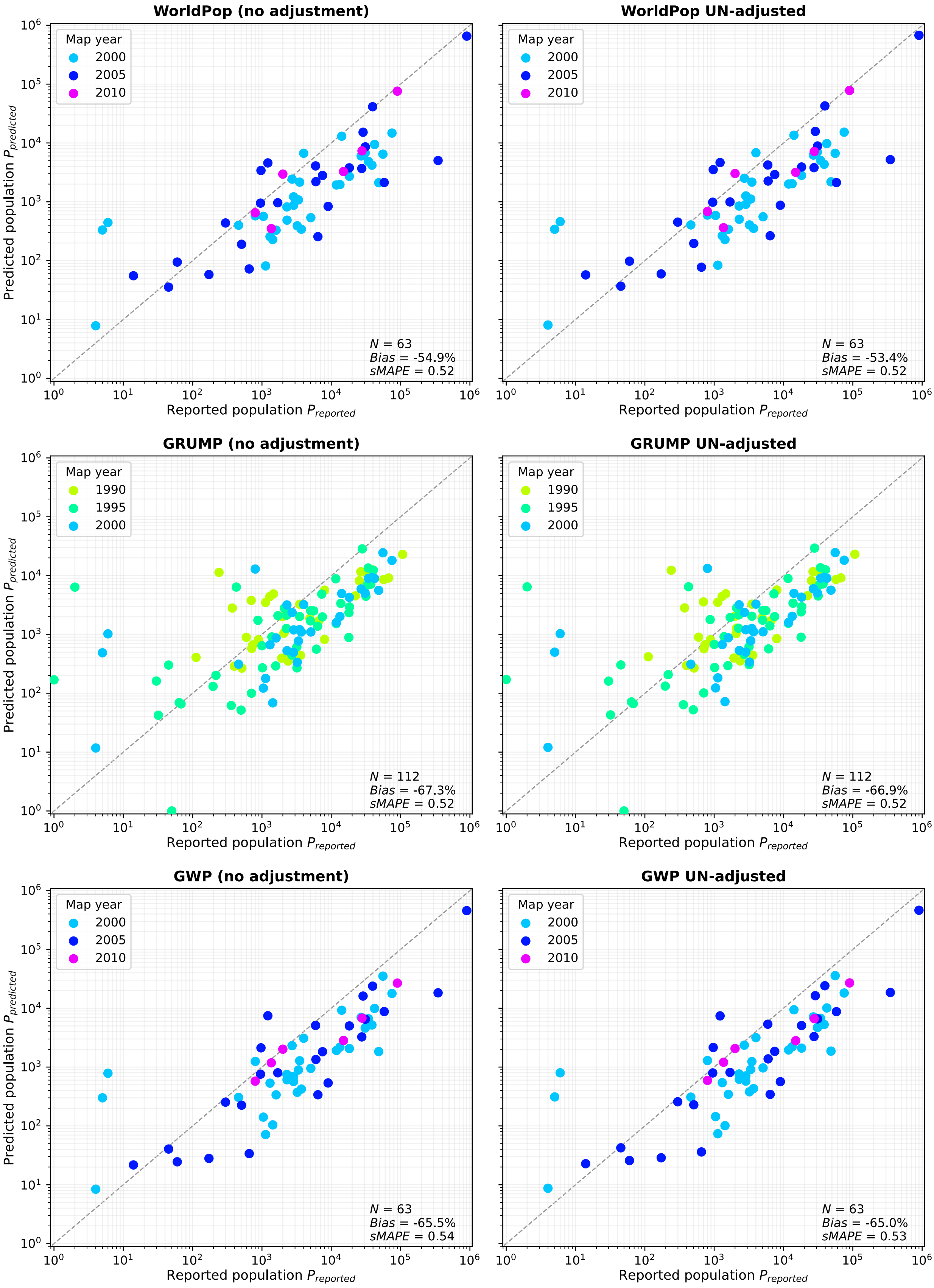

Supplementary Figure 4. Effect of UN-adjustment on population grids. Comparison between unadjusted population datasets (left) and those adjusted using official UN population country statistics (right). No significant differences, with UN-adjusted datasets showing slightly higher population estimates and thus marginally less bias score than their counterparts without adjustment. Therefore, we show only the better performing UN-adjusted datasets in Results.

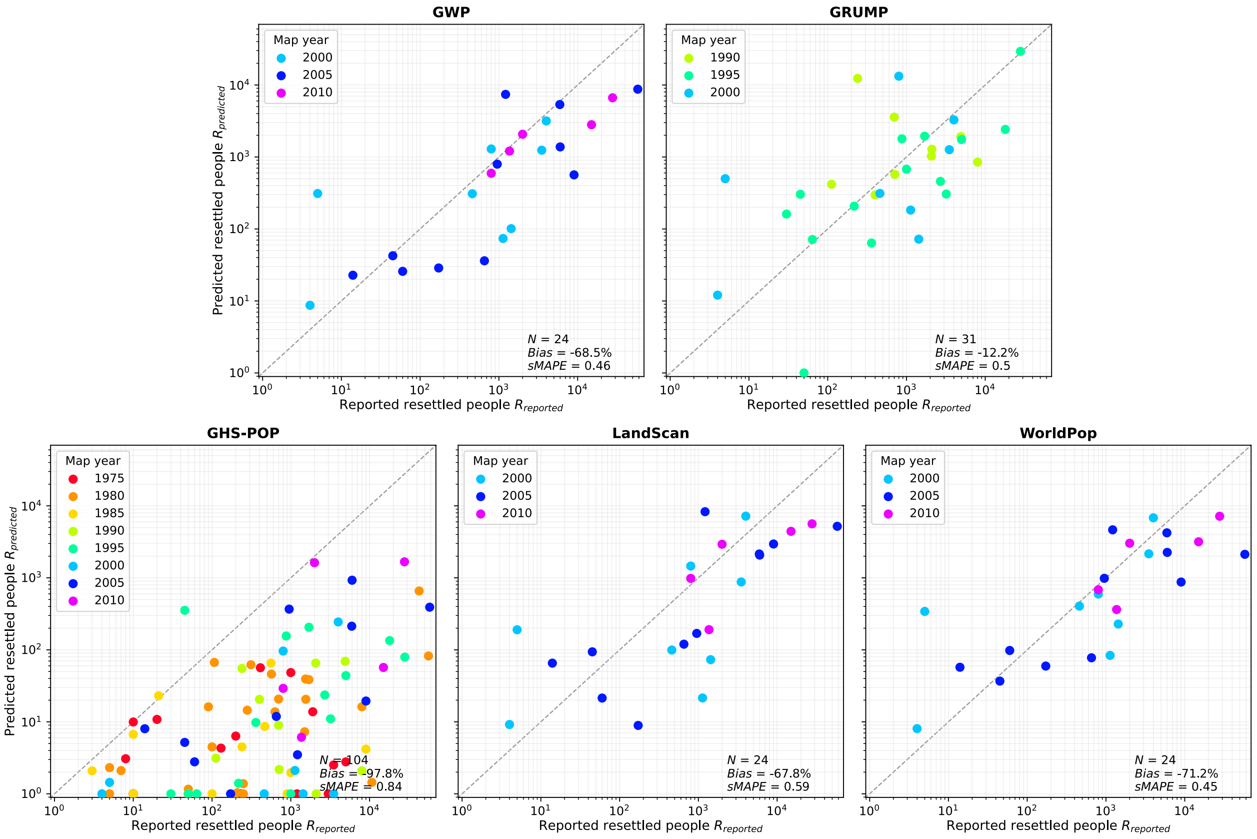

Supplementary Figure 5. Validation scatter plots for the five analysed population datasets (as in Figure 5a–e in the manuscript) but omitting the 203 Chinese reservoirs. Due to the overrepresentation of Chinese reservoirs in the validation data, we tested the robustness of our results when omitting Chinese reservoirs. Results are similar as in the main analysis, except that GRUMP has significantly less bias (–12.2% compared to previously –66.9%) due to a few large Chinese reservoirs with underestimations (see Figure 5b in the manuscript), and WorldPop has more bias (–71.2% compared to previously –53.4%) since it estimated the largest Chinese reservoir very accurately in the main analysis (see Figure 5e in the manuscript). In total, this shows that the results from the main analysis are valid for the global domain despite the overrepresentation of Chinese reservoirs.

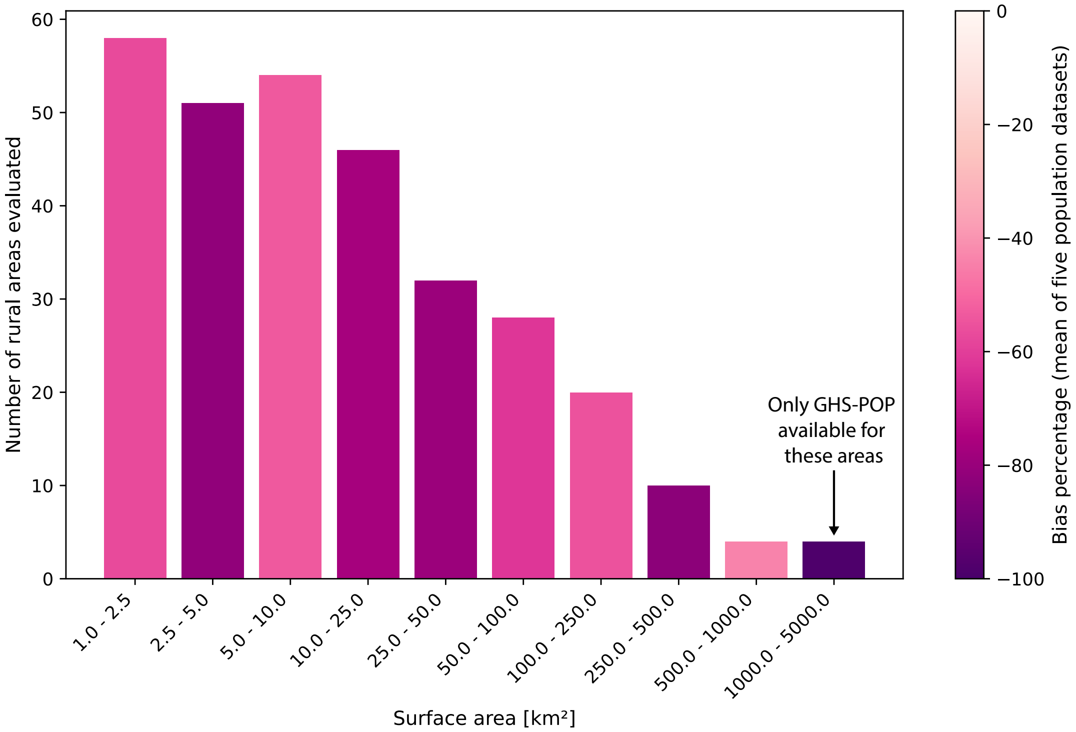

Supplementary Figure 6. Size distribution of evaluated rural areas. Relatively small surface areas (1 – 25 km^2) are most common, but also numerous larger areas up to almost 4000 km^2 in size are included. The colouring of the bars shows the mean of bias percentages of the five population datasets, and it illustrates that area size does not have an influence on the mean bias of the population datasets. The extreme negative bias for the bar at the upper end is due to all polygons in this size group having reference years before 1990, for which only the least accurate dataset GHS-POP is available.

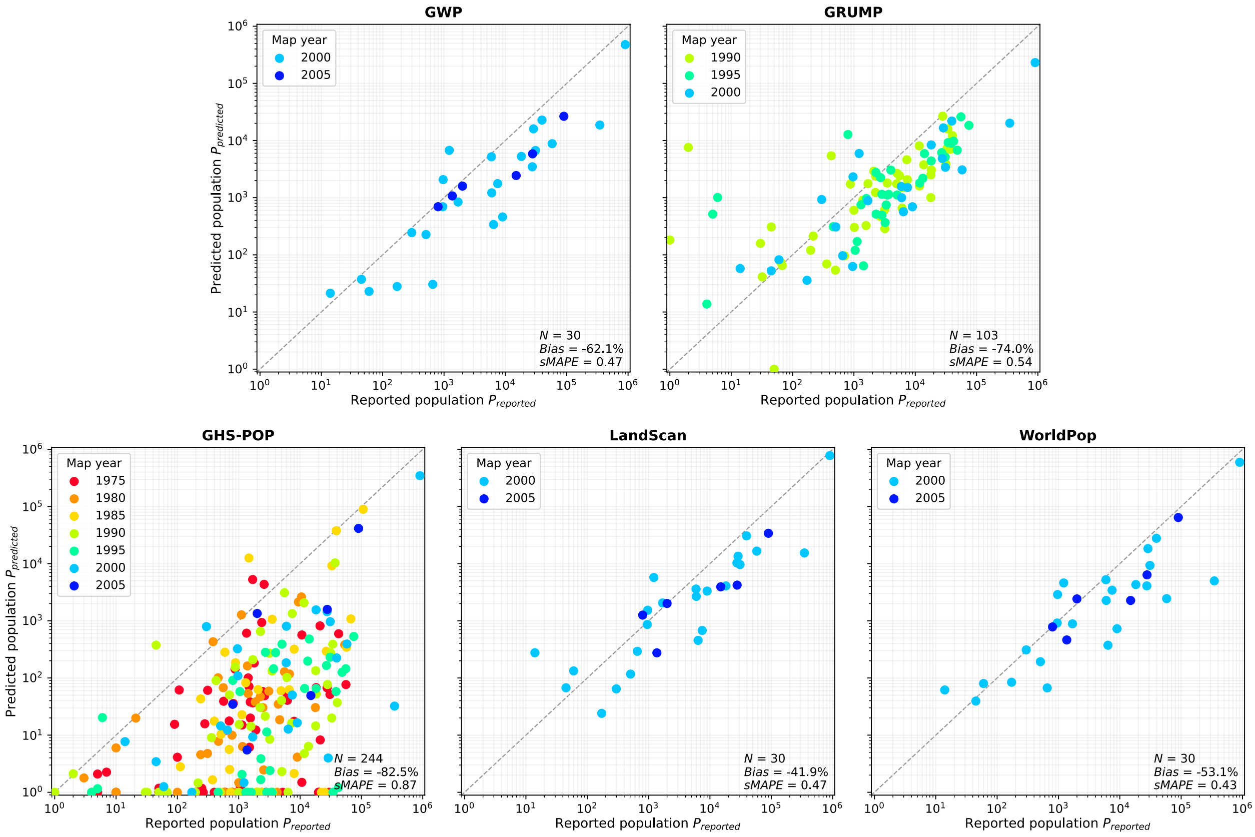

Supplementary Figure 7. Validation scatter plots for the five analysed population datasets (as in Figure 5a–e in the main manuscript), assuming an alternative temporal offset of 10–14 years between population map reference year and year of dam completion, instead of 5–9 years assumed in the main analysis. The results are very similar results, except that LandScan has less bias (–41.9% compared to –68.4% in the main analysis) due to one large underestimating reservoir that dropped out of the analysis due to the change in the temporal offset (see Figure 5d in the manuscript). These results indicates that the temporal offset of 5–9 years assumed in the main analysis is robust.
